# Impact of Gut Recolonization on Liver Regeneration: Hepatic Matrisome Gene Expression after Partial Hepatectomy in Mice

**DOI:** 10.3390/ijms241310774

**Published:** 2023-06-28

**Authors:** Abdul Rahman Amin, Ngatiman M. Hairulhisyam, Raman Nur Fatin Aqilah, Mohd Manzor Nur Fariha, Beth L. Mallard, Fergus Shanahan, Antony M. Wheatley, Muhamad Marlini

**Affiliations:** 1 Department of Basic Medical Science 1, Faculty of Medicine and Health Sciences, Universiti Sains Islam Malaysia, Nilai 71800, Malaysia; aminabdulrahman@usim.edu.my (A.R.A.); hairulhisyam@usim.edu.my (N.M.H.); fatinadqilahrmy@gmail.com (R.N.F.A.); nurfariha@usim.edu.my (M.M.N.F.); 2 Department of Physiology, School of Medicine, University of Galway, H91 TK33 Galway, Ireland; b.mallard@massey.ac.nz (B.L.M.); antony.wheatley@nuigalway.ie (A.M.W.); 3 Alimentary Pharmabiotic Centre, University College Cork, T12 YT20 Cork, Ireland; f.shanahan@ucc.ie

**Keywords:** liver regeneration, matrisome, gut microbiota, germ-free mice, collagen

## Abstract

The hepatic matrisome is involved in the remodeling phase of liver regeneration. As the gut microbiota has been implicated in liver regeneration, we investigated its role in liver regeneration focusing on gene expression of the hepatic matrisome after partial hepatectomy (PHx) in germ-free (GF) mice, and in GF mice reconstituted with normal gut microbiota (XGF). Liver mass restoration, hepatocyte proliferation, and immune response were assessed following 70% PHx. Hepatic matrisome and collagen gene expression were also analyzed. Reduced liver weight/body weight ratio, mitotic count, and hepatocyte proliferative index at 72 h post PHx in GF mice were preceded by reduced expression of cytokine receptor genes *Tnfrsf1a* and *Il6ra*, and *Hgf* gene at 3 h post PHx. In XGF mice, these indices were significantly higher than in GF mice, and similar to that of control mice, indicating normal liver regeneration. Differentially expressed genes (DEGs) of the matrisome were lower in GF compared to XGF mice at both 3 h and 72 h post PHx. GF mice also demonstrated lower collagen expression, with significantly lower expression of *Col1a1*, *Col1a2*, *Col5a1,* and *Col6a2* compared to WT mice at 72 h post PHx. In conclusion, enhanced liver regeneration and matrisome expression in XGF mice suggests that interaction of the gut microbiota and matrisome may play a significant role in the regulation of hepatic remodeling during the regenerative process.

## 1. Introduction

Liver regeneration is a complex process involving multiple cellular factors, cytokines, growth factors, and signaling pathways. The multiple ligands and signaling pathways regulate the events of the regenerative hyperplasia from proliferation of hepatic cells to structural remodeling in order to ensure optimal architecture of the restored liver mass [1,2]. During the regenerative process, the liver goes through three distinctive phases: (a) initiation or priming phase, (b) proliferation phase, and (c) termination phase. These phases occur in chronological order, regulated by manifold ligands and signaling pathways [3].

Many factors have been demonstrated to affect liver regeneration, including gut microbiota. Disturbance of intestinal microbial composition has been shown to affect the regenerative ability of the liver [4]. Mice bred in sterile conditions demonstrate reduced liver regeneration following partial hepatectomy (PHx) [5]. Additionally, gut sterilization with antibiotics produces similar results [6]. Thus, elimination of gut microbiota leads to delayed liver regeneration following PHx.

The role of the gut microbiota in liver regeneration has been attributed to lipopolysaccharide (LPS), the endotoxin derived from Gram-negative bacteria [7,8]. Following PHx, increased permeability of the intestinal barrier allows translocation of LPS in the portal circulation, where it reaches the remnant liver [8]. Active via its TLR4 receptor, LPS directly stimulates Kupffer cells to secrete TNF-α and IL-6, which are the main cytokines involved in priming of hepatocytes. After priming, hepatocytes undergo cell proliferation to restore liver mass. Progression through the cell cycle is mediated by hepatocyte growth factor (HGF), which can also directly stimulate hepatocytes to undergo DNA synthesis and proliferation [9]. Release of ECM-bound HGF and its subsequent activation is mediated by urokinase plasminogen activator (uPA), which is directly increased following PHx [10,11].

Stimulation of the remnant liver following PHx via administration of exogenous endotoxin promotes hepatocyte proliferation, while restriction of endotoxin via administration of antibiotic impairs liver regeneration [6,12]. Furthermore, acute blockade of LPS/TLR4 signaling using anti-TLR4 antibody (MTS510) impairs liver regenerative response in C3H/HeN mice [13]. Mice hyposensitive to LPS due to a *tlr4* gene mutation demonstrate reduced liver regeneration following PHx [13,14].

Apart from cytokines and HGF, the extracellular matrix (ECM) also contributes to liver regeneration. The ECM structural proteins (collagens, proteoglycans, and glycoproteins) and the ECM-associated proteins (ECM-affiliated proteins, ECM regulators, and secreted proteins)—together termed the matrisome—has been suggested to play key roles in all three phases of liver regeneration [15]. During the priming phase of liver regeneration, remodeling of the ECM facilitates release of ECM-bound growth factors such as HGF [10]. During the proliferation and remodeling phases of liver regeneration, remodeling of the ECM facilitates revascularization of hepatocyte clusters and restoration of normal liver architecture [16]. Mabuchi et al. (2004) demonstrated that hepatic stellate cells (HSCs) gathered around hepatocyte clusters, establishing the interaction between HSC–HSC and/or HSC–hepatocyte in the regenerating liver, thus implying the possible role of activated HSC in ECM synthesis during liver regeneration [17].

The ECM also facilitates termination of the regenerative process via ECM–cellular interaction, whereby integrin proteins present on newly synthesized ECM interact with integrin-linked kinase (ILK) complexes present on hepatocytes, leading to suppression of hepatocyte proliferation. Sequestration of cytokines and growth factors such as HGF by newly formed ECM also contributes to termination of the regenerative process [18]. However, the involvement of gut microbiota in liver regeneration with regards to ECM expression has never been elucidated.

The aim of the study was to investigate the role of gut microbiota in liver regeneration, particularly in relation to hepatic matrisome gene expression after PHx. Therefore, liver regeneration and hepatic matrisome gene expression were assessed in GF mice, in GF mice recolonized with normal gut microbiota (XGF mice), and in normal, WT mice. We hypothesized that modified restoration of the hepatic matrisome may play a role in the impaired hepatic regeneration in GF mice. If correct, the reintroduction of gut microbiota would improve hepatic matrisome gene expression following PHx.

## 2. Results

### 2.1. LW/BW Ratio and Liver Growth Percentage

**Figure 1:**
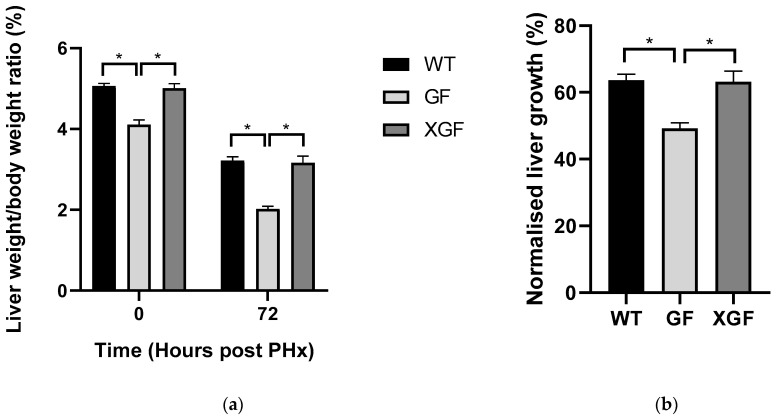
(**a**) Liver weight/body weight ratio and (**b**) normalized liver growth by percentage following PHx in wild-type (WT), germ-free (GF), and ex-germ-free (XGF) mice. Data are presented as means ± SD for n = 3–4 mice per group and time point. * = *p* < 0.05 compared between two mouse groups at a similar time point.

The initial liver mass of GF mice was observed to be smaller compared to normal, WT mice, evident by lower LW/BW at 0 h (Figure 1a). Germ-free mice also demonstrated reduced liver growth and liver mass restoration, evident by lower liver growth percentage and LW/BW at 72 h post PHx (Figure 1). Ex-germ-free mice, on the other hand, demonstrated initial liver size, liver mass restoration, and liver growth that was comparable to controls. These findings, together with an observed higher LW/BW ratio and liver growth percentage in XGF mice compared to GF mice indicated improved liver regeneration in XGF mice.

### 2.2. Proliferative Index

**Figure 2:**
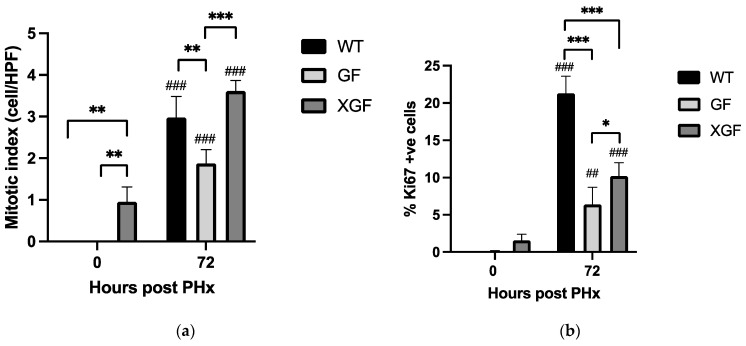
Hepatocyte proliferation following PHx in (**a**) hematoxylin and eosin-stained tissues and (**b**) Ki67-stained liver tissues in wild-type (WT), germ-free (GF), and ex-germ-free (XGF) mice. Data are presented as means ± SD for n = 3–4 mice per group and time point. ## = *p* < 0.01, ### = *p* < 0.001 compared to 0 h after PHx of the same mouse group. * = *p* < 0.05, ** = *p* < 0.01, *** = *p* < 0.001 compared between two mouse groups at a similar time point.

**Figure 3:**
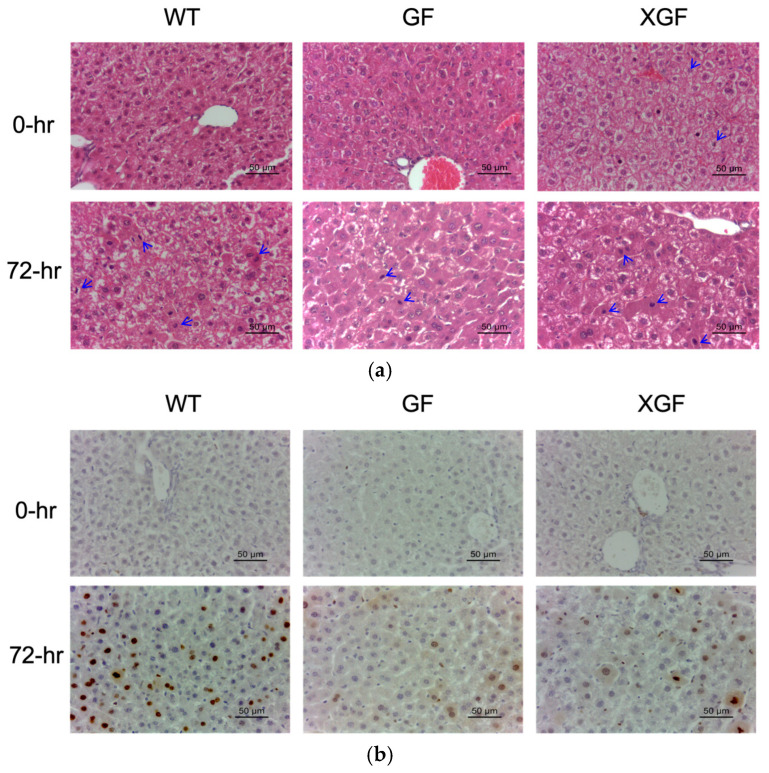
Hepatocyte proliferation following PHx in (**a**) hematoxylin and eosin-stained tissues and (**b**) Ki67-stained liver tissues in wild-type (WT), germ-free (GF), and ex-germ-free (XGF) mice at 0 h and 72 h post PHx. Blue arrows = mitotic figures.

All mice groups demonstrated a significant increase in proliferating hepatocytes at 72 h post PHx (Figure 2 and Figure 3). Hepatocyte proliferation of GF mice was significantly lower compared to WT controls and XGF mice. This was evident in both H&E-stained (Figure 2a and Figure 3a) and Ki67-stained specimens (Figure 2b and Figure 3b). Although demonstrating higher hepatocyte proliferation compared to GF mice, hepatocyte proliferation in XGF mice was still lower compared to WT mice (Figure 2b). Given differences between the GF and control, the WT mice were more evident when observing the proliferative index compared to observation of the LW/BW ratio and liver growth percentage.

### 2.3. Cytokine and Growth Factor Responses

The priming phase of liver regeneration occurs during the first 5 h following PHx and is important in initiating liver regeneration of the remnant liver [11]. It is characterized by a cytokine response, mainly TNF-α and IL6. Here, we assessed the local response of the remnant hepatic tissue at 3 h post PHx via expression of *Tnfrsf1a* and *Il6ra* genes, which are genes encoding TNFR1 and IL6R proteins, respectively.

**Figure 4:**
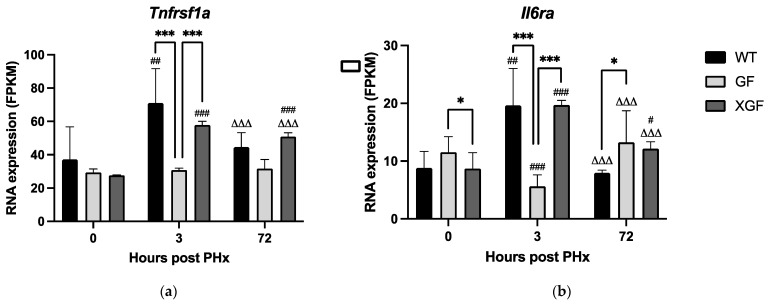
RNA expression of (**a**) *Tnfrsf1a* and (**b**) *Il6ra* following PHx in WT, GF, and XGF mice. Data are presented as means ± SD, for n = 3–4 mice per group and time point. # = *p* < 0.05, ## = *p* < 0.01, ### = *p* < 0.001 compared to 0 h after PHx of the same mouse group. ∆∆∆ = *p* < 0.001 compared to the same mouse group at different time points (3 vs. 72 h after PHx). * = *p* < 0.05, *** = *p* < 0.001 compared to different mouse groups at a similar time point.

**Figure 5:**
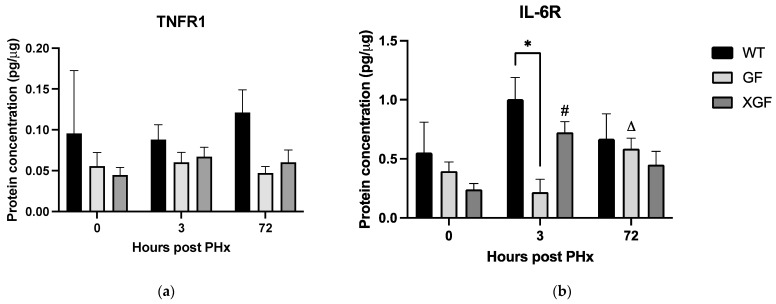
Liver tissue concentration of (**a**) TNFR1 and (**b**) IL-6RA following PHx in WT, GF, and XGF mice. Data are presented as means ± SD, for n = 3–4 mice per group and time point. # = *p* < 0.05 compared to 0 h after PHx of the same mouse group. ∆ = *p* < 0.05 compared to the same mouse group at different time points (3 vs. 72 h after PHx). * = *p* < 0.05 compared to different mouse groups at a similar time point.

Expression of *Tnfrsf1a* and *Il6ra* at 3 h post PHx in GF mice was significantly reduced compared to controls (*Tnfrsf1a*, *p* = 0.0006; *Il6ra*, *p* = 0.0006) (Figure 4). The reduced *Il6ra* expression was reflected in significantly reduced IL6RA protein concentration at 3 h post PHx in GF mice compared to controls (*p* = 0.0338) (Figure 5b). Expression of *Tnfrsf1a* and *Il6ra* in XGF mice at 3 h post PHx was similar to controls (Figure 4). There were no significant differences in tissue concentration of TNFR1 between the three mouse groups at any of the time points assessed (Figure 5a).

**Figure 6:**
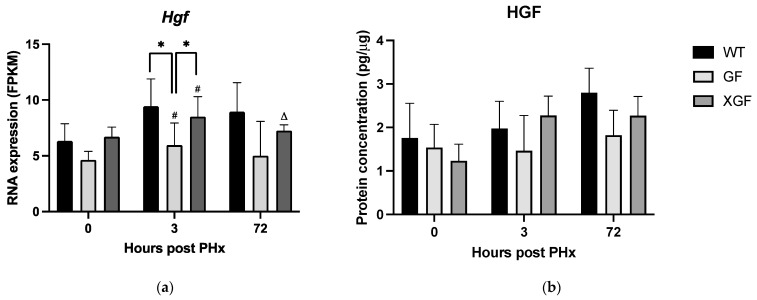
(**a**) RNA expression of *Hgf* gene and (**b**) liver tissue concentration of HGF protein following PHx in WT, GF, and XGF mice. Data are presented as means ± SD, for n = 3–4 mice per group and time point. # = *p* < 0.05 compared to 0 h after PHx of the same mouse group. ∆ = *p* < 0.05 compared to the same mouse group at different time points (3 vs. 72 h after PHx). * = *p* < 0.05 compared to different mouse groups at a similar time point.

Expression of *Hgf*, the gene encoding hepatocyte growth factor (HGF), was also assessed. HGF is responsible for initiation of DNA synthesis and progression of the hepatocyte through the cell cycle during cell proliferation. At 3 h post PHx, expression of *Hgf* in GF mice was significantly lower compared to controls (*p* = 0.0047) and XGF mice (*p* = 0.011) (Figure 6a). Expression of *Hgf* in XGF mice at 3 h post PHx was similar to controls. Accordingly, there was a trend of lower tissue concentration of HGF at 3 and 72 h post PHx in GF mice compared to WT and XGF mice (Figure 6b).

### 2.4. Expression of Matrisome Genes

**Figure 7:**
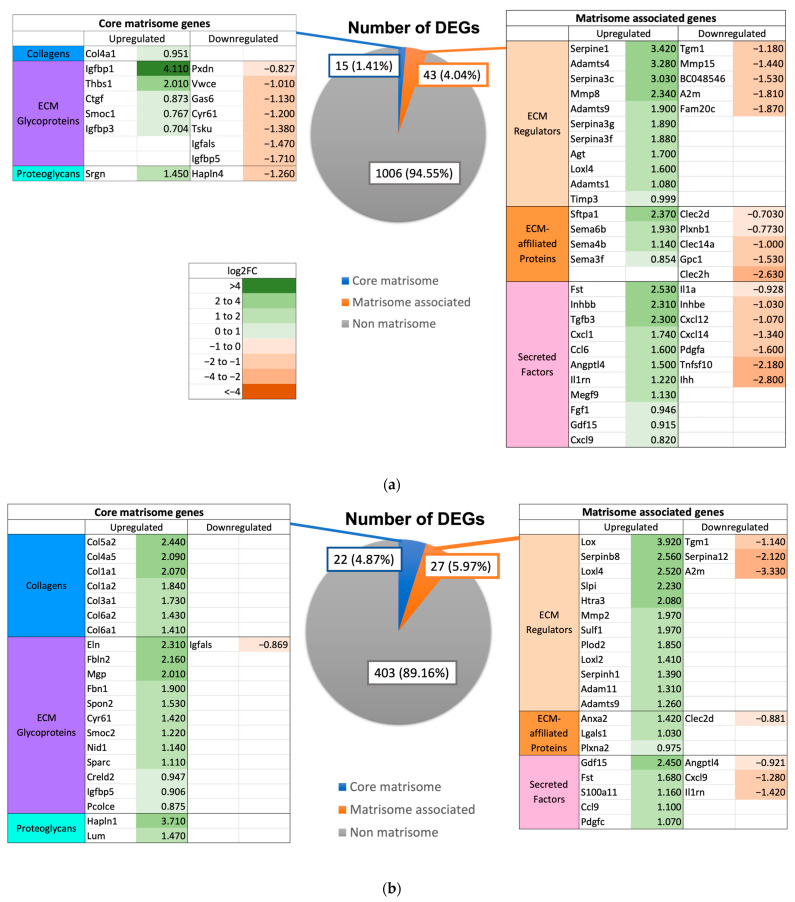
Number of DEGs at (**a**) 3 h (0 h vs. 3 h) and (**b**) 72 h (0 h vs. 72 h) post PHx in wild-type mice. Listed in tables are the DEGs, values in green represent positive log2FC indicating upregulation and values in red represent negative log2FC indicating downregulation.

Expression of matrisome genes was observed in the different mouse groups. The 0 h time point was used as a control to identify differentially expressed genes (DEGs) of the matrisome at 3 h and 72 h post PHx in the respective groups. At 3 h post PHx in WT mice, a total of 33 DEGs were upregulated and 25 DEGS were downregulated (total 58 DEGs). At 72 h post PHx, 49 DEGs were identified (41 upregulated and 8 downregulated) (Figure 7). Notable upregulated DEGs include ECM regulators *Serpine1* (log2FC = 3.42, *p* = 0.0012) and *Mmp8* (log2FC = 2.34, *p* = 0.0072) at 3 h post PHx and *Lox* (log2FC = 3.92, *p* = 0.0023), *Serpinb8* (log2FC = 2.56, *p* = 0.0023), and *Loxl4* (log2FC = 2.52, *p* = 0.0023) at 72 h post PHx.

**Figure 8:**
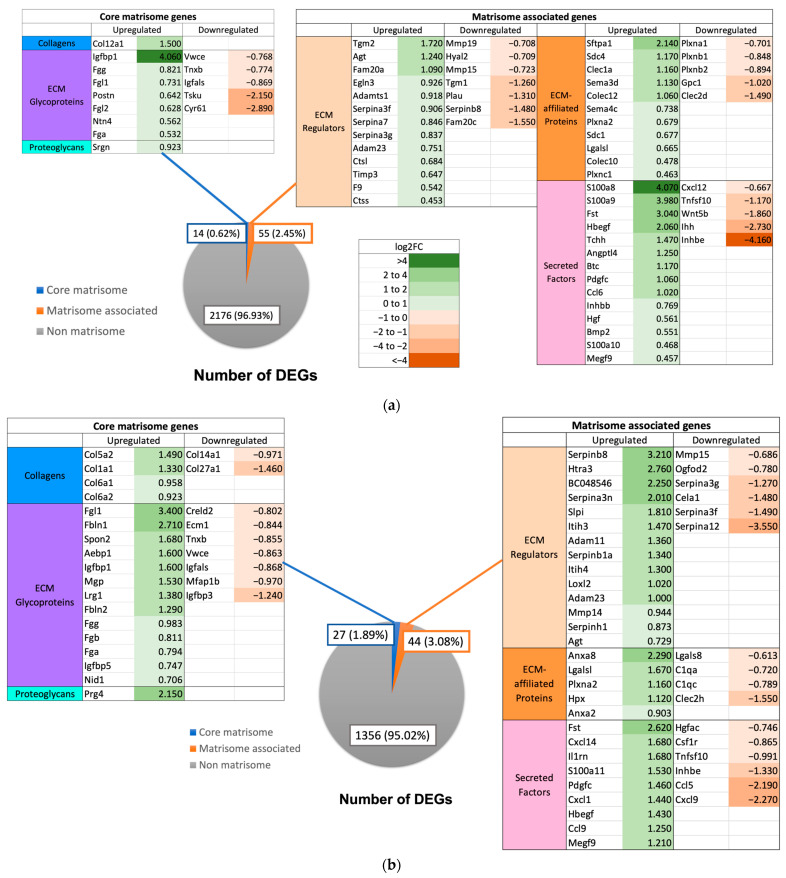
Number of DEGs at (**a**) 3 h (0 h vs. 3 h) and (**b**) 72 h (0 h vs. 72 h) post PHx in germ-free mice. Listed in tables are the DEGs, values in green represent positive log2FC indicating upregulation and values in red represent negative log2FC indicating downregulation.

**Figure 9:**
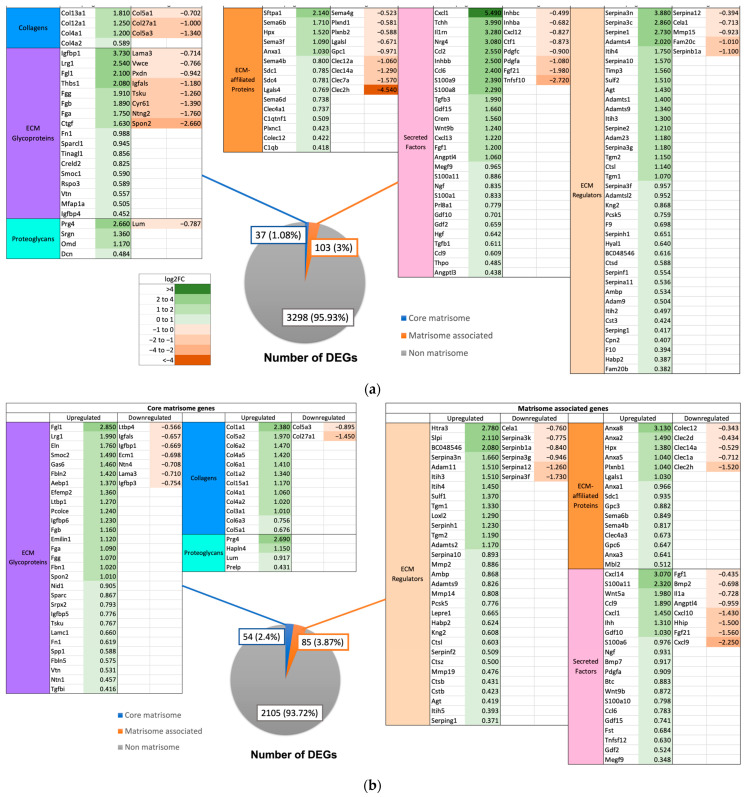
Number of DEGs at (**a**) 3 h (0 h vs. 3 h) and (**b**) 72 h (0 h vs. 72 h) post PHx in ex-germ-free mice. Listed in tables are the DEGs, values in green represent positive log2FC indicating upregulation and values in red represent negative log2FC indicating downregulation.

In GF mice, a total of 69 (47 upregulated and 22 downregulated) and 71 (46 upregulated and 25 downregulated) DEGs were identified at 3 h and 72 h post PHx, respectively (Figure 8). Ex-germ-free mice demonstrated a total of 140 (106 upregulated and 34 downregulated) and 139 (111 upregulated and 28 downregulated) DEGs at 3 h and 72 h, respectively (Figure 9). All mouse groups demonstrated a higher number of upregulated core matrisome genes at 72 h post PHx, indicating increased distribution of ECM structural proteins for hepatic remodeling.

**Figure 10:**
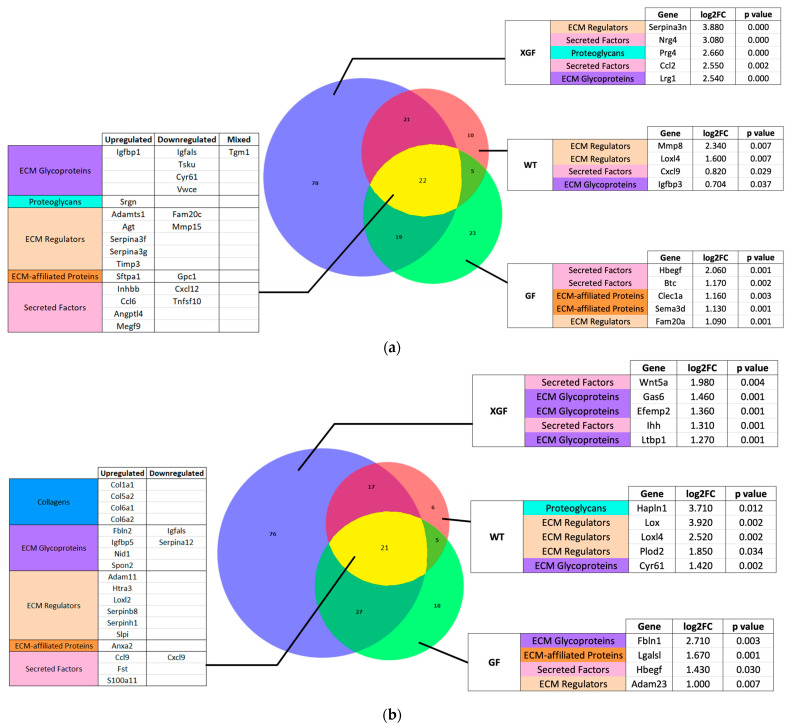
Venn diagram of number of DEGs in wild-type (WT), germ-free (GF), and ex-germ-free (XGF) mice at (**a**) 3 h and (**b**) 72 h post PHx. Overlapping sets show common DEGs between the groups. Listed are the commonly expressed genes between mouse groups and highly expressed unique genes from each group.

**Figure 11:**
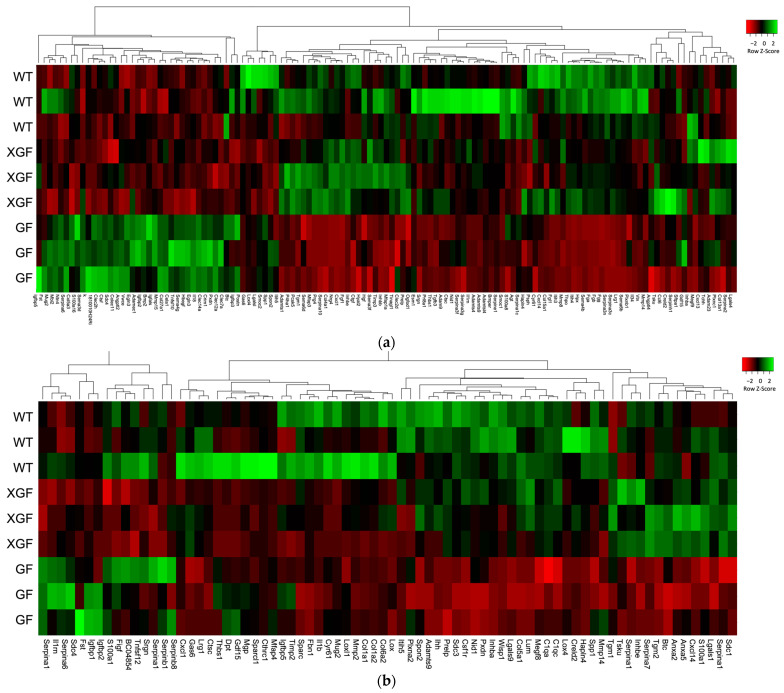
Heatmap representation of matrisome gene expression in wild-type (WT), germ-free (GF), and ex-germ-free (XGF) mice at (**a**) 3 h and (**b**) 72 h post PHx with hierarchal clustering of genes (columns) and samples (rows). Green in the heatmap indicates upregulation, while red indicates downregulation.

Comparison of the matrisome DEGs between the different mouse groups showed that a total of 22 and 21 DEGs were similarly expressed by all three mouse groups at 3 h and 72 h post PHx, respectively (Figure 10). Notable commonly upregulated matrisome DEGs were ECM regulators *Adamts1*, *Agt*, *Serpina3f*, *Serpina3g*, and *Timp3* at 3 h, and *Adam11*, *Htra3*, *Loxl2*, *Serpinb8*, and *Serpinhl* at 72 h post PHx. Clustering of samples using ANOVA showed that XGF and WT mice had similar matrisome gene expression patterns at both 3 h and 72 h post PHx (Figure 11). The matrisome gene expression pattern of GF mice samples was distinct from the other groups at 3 h and 72 h post PHx.

### 2.5. Expression of Collagen Genes

**Figure 12:**
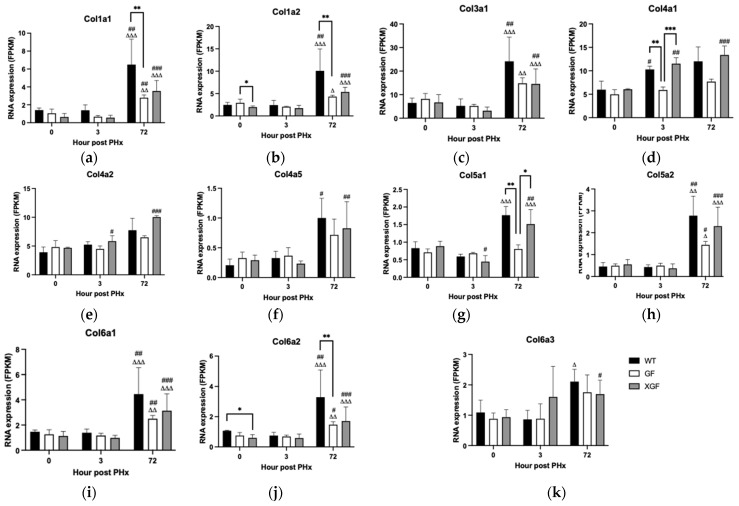
RNA expression of (**a**) *Col1a1*, (**b**) *Col1a2*, (**c**) *Col3a1*, (**d**) *Col4a1*, (**e**) *Col4a2*, (**f**) *Col4a5*, (**g**) *Col5a1*, (**h**) *Col5a2*, (**i**) *Col6a1*, (**j**) *Col6a2*, and (**k**) *Col6a3* following PHx in WT, GF, and XGF mice. Data are presented as means ± SD, for n = 3–4 mice per group and time point. # = *p* < 0.05, ## = *p* < 0.01, ### = *p* < 0.001 compared to 0 h after PHx of the same mouse group. ∆ = *p* < 0.05, ∆∆ = *p* < 0.01, ∆∆∆ = *p* < 0.001 compared to the same mouse group at different time points (3 vs. 72 h after PHx). * = *p* < 0.05, ** = *p* < 0.01, *** = *p* < 0.001 compared to different mouse groups at a similar time point.

**Figure 13:**
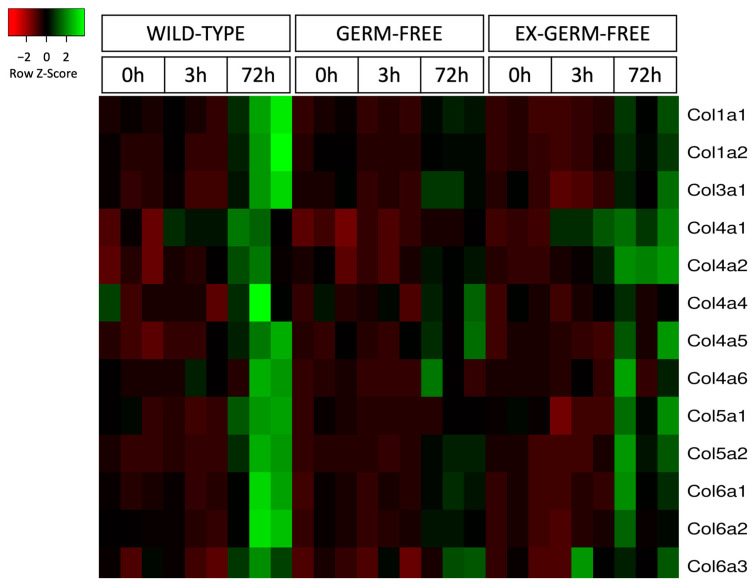
Heatmap representation of the expression levels of main collagen genes following PHx in wild-type, germ-free, and ex-germ-free mice. Expression data are fragments per kilobase of transcript per million fragments mapped (FPKM) reads for n = 3–4 mice per group and time point. Green in the heatmap denotes upregulation, while red denotes downregulation.

Upregulation of collagen genes was observed at 72 h post PHx, which coincides with the early remodeling phase of liver regeneration. The remodeling phase is characterized by increased production of ECM structural proteins, mainly collagen, for restoration of normal hepatic architecture. All mouse groups demonstrated significant upregulation of collagen genes at 72 h post PHx, which include *Col1a1*, *Col1a2*, *Col3a1*, *Col4a5*, *Col5a2*, *Col6a1*, and *Col6a2* in WT mice, *Col1a1*, *Col5a2*, *Col6a1*, and *Col6a2* in GF mice, and *Col1a1*, *Col1a2*, *Col3a1*, *Col4a1*, *Col4a2*, *Col4a5*, *Col5a1*, *Col5a2*, *Col6a1*, *Col6a2*, and *Col6a3* in XGF mice (Figure 12). Expression of *Col1a1* (*p* = 0.0028), *Col1a2* (*p* = 0.0028), *Col5a1* (*p* = 0.0028), and *Col6a2* (*p* = 0.0028) (Figure 12a,b,g,j) was significantly lower in GF mice compared to WT mice at 72 h post PHx. The heatmap representation of collagen gene expression between the different groups demonstrates the highest expression of collagen genes in WT mice at 72 h post PHx (Figure 13).

## 3. Discussion

### 3.1. Reduced Liver Mass Restoration, Hepatocyte Proliferation, and Immune Response in GF Mice

In this study, we confirmed reduced liver mass restoration following PHx in GF mice, evident by the lower LW/BW ratio and liver growth percentage at 72 h post PHx. As liver mass restoration is contributed to by hypertrophy and proliferation of hepatocytes, reduced liver mass restoration in GF mice is suggested due to its reduced hepatocyte proliferation, as observed in this study. Further investigation of hepatocyte proliferation mechanisms reveals reduced immune response in GF mice, evident by reduced cytokine TNF-α and IL6 receptor responses. Immune response following PHx is known to regulate hepatocyte proliferation, whereby TNF-α and Il6 secreted by Kupffer cells prime hepatocytes in preparation to exit their dormant phase and enter the cell cycle to proliferate.

Gnotobiotic mice have been shown to have an underdeveloped immune system due to the lack of interplay between the commensal microorganism and the host immune system [19]. Gut microbiota also plays a role in immune response following antigen exposure [20]. Our findings indicating reduced liver regeneration in GF mice were in accordance with previous studies, where reduced liver regeneration was observed following PHx in mice with diminished LPS/TLR4 signaling. The reduced liver regeneration was demonstrated in GF, and antibiotic-treated mice [5,21], and in LPS-hyposensitive, C3H/HeJ mice [13,14]. Therefore, our findings reveal a further implication of an underdeveloped immune system of the sterile mouse, and the importance of gut microbiota.

Taken together, these findings suggest that the reduced liver regeneration following PHx in GF mice may be due to an impaired immune response. While highlighting the role of the immune response in liver regeneration, we cannot exclude the fact that liver regeneration occurred in GF mice, albeit at a reduced capacity, as shown from our findings. Therefore, the role of the immune response pathway in the overall complex process of liver regeneration—whether as a crucial pathway, or merely an auxiliary one—must be further studied.

### 3.2. Improved Liver Regeneration in XGF Mice

To assess the role of gut microbiota in liver regeneration, we observed liver regeneration in GF mice that have been reconstituted with normal gut microbiota. Compared to GF mice, XGF mice demonstrated increased liver mass restoration, hepatocyte proliferation, and cytokine response, indicating an overall improved liver regeneration post PHx. Improved liver regeneration in XGF mice may be attributed to the re-establishment of gut microbiota allowing secreted LPS to reach the remnant liver following PHx. LPS acting on its TLR4 receptor induces Kupffer cells to secrete TNF-α and IL-6 and initiate the regenerative response [7,8]. Subsequent hepatocyte proliferation and liver mass restoration therefore proceeds normally following a normal cytokine response, as demonstrated in our current study. This is consistent with previous studies, where inhibition of LPS/TLR4 signaling leads to impaired liver regeneration [13]. It has been demonstrated that colonization of sterile gut with normal gut microbiota promotes physiological and structural functions of the intestinal barrier, which further contributes to the intestinal homeostasis [22]. As LPS originates from intestinal bacteria, normal intestinal homeostasis and normal intestinal wall function are important in allowing a proper LPS response following PHx.

Improved liver regeneration in XGF mice may also be due to an improved immune response in these mice, evident by the increased cytokine TNF-α and IL-6 receptor responses. The introduction of gut microbiota may promote immune system development in these previously sterile mice, which are known to have an underdeveloped immune system. While assessment of the immune system of these mice prior to PHx was not assessed, phenotypical changes were observed in XGF mice in the form of increased liver mass. The LW/BW ratio of XGF mice prior to PHx was similar to the control mice and significantly higher than that of their sterile counterpart, therefore further demonstrating physiological and structural improvements in XGF mice.

Taken together, these findings suggest that the improved liver regeneration in XGF mice may be due to re-establishment of normal gut microbiota and subsequent re-establishment of the immune response following PHx, in addition to the improved immune system of XGF mice.

### 3.3. Role of Cytokine Receptors in Liver Regeneration

The role of cytokines in liver regeneration have been extensively studied, especially the cytokines IL-6 and TNF-α [1,23]. Our study has further established the role of IL-6 and TNF-α by demonstrating upregulation of genes responsible for the receptors of these cytokines. These are the *Il6ra* gene, which encodes for the IL-6 receptor Il-6RA, and *Tnfrsf1a*, which encodes for the TNF-α receptor TNFR1.

In both WT and XGF mice, upregulation of *Tnfrsf1a* and *Il6ra* occurred at 3 h post PHx, coinciding with the priming phase of liver regeneration. During the proliferative phase, at 72 h post PHx, both *Tnfrsf1a* and *Il6ra* genes returned to baseline levels in both mouse groups. Interestingly, genes encoding the cytokines themselves were not upregulated, suggesting that increased TNF-α and IL-6 levels occur systematically, and that the liver tissue response is to upregulate the receptors to capture the incoming cytokines. Accordingly, increased TNF-α and IL-6 plasma levels following PHx is an established phenomenon [24,25]. The local tissue response, however, is not well studied. Therefore, our findings of upregulation of receptors in the hepatic tissue further enhance our understanding of the role of cytokines and their receptors during liver regeneration.

Germ-free mice, however, showed no significant upregulation of both *Tnfrsf1a* and *Il6ra* at 3 h post PHx, while at 72 h post PHx, *Il6ra* was significantly upregulated compared to the 3 h time point. The absence of *Tnfrsf1a* and *Il6ra* upregulation during the priming phase may indicate a delayed cytokine response in this mouse group. An increase in TNF-α and IL-6 plasma levels post PHx in GF mice may only occur at a later stage, suggested by the upregulation of *Il6ra* at 72 h post PHx. The delayed cytokine response, together with subsequent reduced liver growth in absence of gut microbiota, further establishes the role of cytokines in liver regeneration.

### 3.4. Role of Matrisome in Liver Regeneration

Our study demonstrated changes in matrisome expression during liver regeneration. Changes in ECM components have been shown to occur throughout liver regeneration, starting from the priming phase [26,27]. However, changes in the ECM-associated proteins have been less studied. In our study, observed changes in the expression of ECM structural components and ECM-associated proteins occur at both the priming phase (3 h post PHx) and proliferation/remodeling phase (72 h post PHx) of liver regeneration, which is in accordance with the proposed role of the matrisome in re-establishment of normal hepatic structure after PHx.

Expressed genes during the priming phase were that of ECM regulators involved in ECM degradation. This includes the family of proteolytic enzymes such as ADAMTS and MMPs, and their regulatory inhibitors such as TIMPs and Serpins, which regulate proteolytic activity. Degradation of hepatic ECM leads to release of ECM-bound hepatocyte growth factor (HGF), a known mitogen for hepatocytes, and therefore, ECM plays an important role for hepatocyte proliferation [9,24]. This was in accordance with previous studies demonstrating increased expression of proteases and their regulatory inhibitors during the priming phase of liver regeneration [10,28,29,30].

It was also observed that expression of ECM structural proteins (core matrisome component) was minimal during the priming phase. However, during the remodeling phase, there was a significant increase in expression of ECM structural proteins, especially collagen. The ECM structural proteins play an important role in hepatic remodeling and restoration of normal hepatic architecture during the remodeling phase, most likely via activated HSC [16,17]. Our findings of increased collagen expression in the mouse groups at 72 h post PHx is therefore in accordance with the literature. There was also increased expression of ECM regulators responsible for ECM stabilization, such as the LOX family of proteins. The LOX family of enzymes contributes to covalent crosslinking between collagen and elastin, which is important for stabilizing matrix components and contributing to the tensile strength and structural integrity of tissues [31,32].

Taken together, our findings indicate the dynamic role played by the matrisome during liver regeneration. Both ECM-associated proteins and structural ECM proteins are expressed appropriately according to the requirement of the regenerating liver.

### 3.5. Matrisome Expression in GF and XGF Mice

Germ-free mice demonstrated a distinct matrisome expression pattern compared with WT mice. It was observed that in GF mice there was a lower percentage of upregulated DEGs and a higher percentage of downregulated DEGs at 72 h post PHx compared with the WT mice. Collagen expression at 72 h post PHx was also significantly lower in the GF mice compared with the WT mice. This suggests that reduced liver regeneration in GF mice is also associated with reduced matrisome and collagen expression during the remodeling phase.

Matrisome expression in XGF mice was similar to mice at both 3 h and 72 h post PHx. There were also no significant differences in collagen expression of XGF and WT mice at 72 h post PHx. The similar pattern of matrisome gene expression of XGF to WT mice indicate the role of gut microbiota in liver regeneration. As XGF mice were previously sterile, reintroduction of gut microbiota resulted in an improved matrisome gene expression of XGF mice to become similar to that of WT mice.

Although XGF mice demonstrated improved matrisome expression, differences between XGF and WT mice were still present. These included the higher number of DEGs in XGF mice at 3 h and 72 h post PHx compared with the WT mice. Comparison between the two groups also demonstrated matrisome genes that were exclusively expressed in either XGF or WT mice at 3 h and 72 h post PHx. Furthermore, the heatmap representation of collagen genes showed a degree of higher collagen gene expression in WT mice compared to XGF mice. This could be due to dissimilarity in the gut microbial composition between the two groups, as the gut microbiota in XGF may not have fully equilibrated with that of the WT mice. Normalization of gut microbiota following reconstitution has been reported to take from one week to more than 20 days [33]. Changes in hepatic expression following introduction of microbiota in GF mice are observed to vary according to the composition of the administered microbiota [34,35,36]. In addition, GF mice are known to have impaired intestinal barrier structure and function [22]. These differences may account for the different matrisome gene expression and regenerative response in the WT and XGF mice in our study.

## 4. Material and Methods

### 4.1. Animals

Male mice, 8–10 weeks old, were used in the study. Wild-type mice of the C57BL/6 strain were acquired from Charles River, UK, and were housed in a temperature and humidity-controlled room under a 12 h light–dark cycle and maintained on a standard mouse diet with free access to water. Germ-free mice (C57BL/6 background) were bred in the BSU (Biological Service Unit), University College Cork (UCC), Ireland. All GF mice were kept in a gnotobiotic facility and were housed in flexible plastic isolators. Germ-free mice were given autoclaved food and sterile tap water. Regular fecal monitoring was performed to ensure that they remained free from all bacteria, exogenous viruses, fungi, and parasites. Recolonization of GF mice with normal gut microbiota was performed by exposing GF mice with fecal material from normal WT mice for one week, as mice are coprophagic (fecal eating) animals.

### 4.2. Animal Ethics

All experiments were undertaken in accordance with Irish Medicine Board Acts 1995 and 2006, Project Authorization numbers AE19125/P050 and AE19130/P038, and the Animal Care Research Ethics Committee (ACREC), University of Galway, and the Animal Experimental Ethics Committee (AEEC), University College Cork, application number 15/DEC/02. Universiti Sains Islam Malaysia (USIM) ethical approval was obtained from *Jawatankuasa Etika Haiwan* (JKEH) USIM (Reference number USIM/AEC/AUP/2018(1)).

### 4.3. Anesthetic Protocols

Wild-type mice and XGF mice underwent a 70% PHx or sham laparotomy under inhalational isoflurane anesthesia. Isoflurane was administered to the mice at a concentration of 4% in an induction chamber and was maintained at 1.5–2.0% during the surgery. Isoflurane was vaporized in a continuous 500–1000 mL oxygen flow. The core temperature was continuously monitored throughout the procedure. The level of anesthesia was assessed by monitoring the respiratory rate, righting reflex, and pedal withdrawal in response to pain stimulus. Partial hepatectomy or sham laparotomy in GF mice was performed under Ketamine (100 mg/kg)/Xylazine (10 mg/kg) anesthesia (i.p.) under sterile conditions.

### 4.4. Partial Hepatectomy and Sampling of Tissues

Partial hepatectomy in WT mice was performed in the Microcirculation Research Laboratory (MRL), University of Galway. Partial hepatectomy in GF mice was performed in the flexible plastic isolators at the BSU, UCC. Partial hepatectomy in XGF mice was also performed in the BSU, UCC.

Mice underwent a two-thirds PHx with removal of the right medial, left medial, and left lobes, which constitute 70% of the total liver volume. Control (0 h) mice underwent gentle manipulation of liver lobes without excision (sham operation) and were killed immediately after the sham operation. Sampling of the remnant liver tissue was done at 3, and 72 h following initial surgery.

Samples for molecular studies were collected and kept in RNAlater (Sigma, St. Louis, MO, USA). Liver tissues were fixed in 10% formalin solution and embedded in paraffin for histology studies. All samples were shipped to the Faculty of Medicine and Health Sciences, Universiti Sains Islam Malaysia, at either 4 °C or −20 °C (World Courier, Dublin, Ireland).

### 4.5. Liver Weight/Body Weight Ratio (LW/BW)

Liver mass restoration was estimated from the animal’s LW/BW ratio at 0 h and 72 h time points. The regenerating liver was removed en bloc, and the liver weight was measured. The LW/BW ratio was calculated as: LW/BW ratio (%) = 100 × (regenerating liver weight/body weight). The percentage of liver growth was then calculated from the LW/BW ratio.

### 4.6. Histology

#### 4.6.1. Hematoxylin and Eosin Staining, and Mitotic Count

Paraffin-embedded liver tissues underwent preparation and were subsequently stained with hematoxylin and eosin stain as per standard protocols. Hepatocyte mitotic activity was assessed using H&E-stained liver sections under light microscopy. Mitotic figures were counted in 25–30 high-power fields (HPF; ×400 magnification) in each tissue section. Investigators were blinded to mouse group time after PHx. Data were expressed as mitotic figures/HPF. Four to six tissue sections were examined for each time point and animal group.

#### 4.6.2. Ki67 Immunohistochemical Staining and Proliferative Index

Tissue slides were deparaffinized and rehydrated prior to an antigen retrieval process, whereby tissue slides were heated in sodium citrate buffer. This was followed by incubation in methanol 0.3% hydrogen peroxide and bovine serum albumin (BSA) for deactivation of endogenous peroxidase activity and blocking of nonspecific tissue proteins, respectively. Incubation with primary Ki67 monoclonal antibodies (Clone SP6, Novus Biologicals, Centennial, CO, USA) at 4 °C overnight was followed by incubation with biotinylated polyclonal anti-rabbit antibodies (Novus Biologicals, USA) as secondary antibodies. Elite ABC kits (Vektor Laboratories, Newark, CA, USA) were used to enhance immunoreactivity. Subsequent chromogen staining using diaminobenzidine hydrogen peroxide (Sigma, St. Louis, MO, USA) and hematoxylin counterstain was employed. Randomized HPF was selected from each group for proliferative index counting. The proliferative index was counted as number of Ki67-positive cells out of 100 cells.

### 4.7. Protein Assay

Protein extraction was done using the Tissue Protein Extraction Reagent (T-PER) (Thermo Scientific, Waltham, MA, USA) according to the manufacturer’s protocol. For protein assay, the mouse premixed multi-analyte kit (R&D Systems, Inc., Minneapolis, MN, USA) was used according to the manufacturer’s protocol. The Luminex MAGPIX analyzer was used to read the microparticle beads.

### 4.8. Statistical Analysis

All results were expressed as mean values ± standard deviation (SD). Data were analyzed using the Mann–Whitney test and the Kruskal–Wallis one-way analysis of variance (ANOVA) with Dunn’s multiple comparisons test using GraphPad Prism for Windows (Version 5.00, GraphPad Software, San Diego, CA, USA). Two-way ANOVA with Tukey’s multiple comparisons test was used in the analysis of mitotic index and percentage of Ki67-positive cells. A *p*-value of <0.05 was considered statistically significant.

### 4.9. RNA Sequencing and Analysis

#### 4.9.1. RNA Sequencing

RNA extraction was done using the RNeasy mini kit (Qiagen, Hilden, Germany) according to the manufacturer’s protocol. The RNA integrity number (RIN) was evaluated using the Agilent 2100 Bioanalyzer (Agilent, CA, USA), and samples with RIN between 8 and 10 were selected for sequencing. Library preparation was done using a TruSeq RNA Sample Preparation Kit (Illumina, CA, USA). Sequencing was performed using the Illumina HiSeq 2500 system (Illumina, CA, USA).

#### 4.9.2. Transcriptome Data Analysis

Filtered reads were mapped to the reference genome related to the species using TopHat, and gene expression level was measured with Cufflinks v2.1.1 [37,38]. Analysis of differential expression was performed by Cuffdiff [39]. DEGs were identified on the q-value threshold less than 0.05 for correcting errors by multiple testing [40]. Genes were classified according to biological process, cellular component, and molecular function using the gene ontology (GO) database. The GO-based trend test was performed using the Fisher’s exact test to characterize the identified genes. Selected genes of *p*-values < 0.001 were regarded as statistically significant.

## 5. Conclusions

This study has demonstrated that reintroduction of gut microbiota in GF mice improves liver regeneration following PHx, indicating a role of gut microbiota in liver regeneration. However, whether gut microbiota is truly significant for liver regeneration remains a question. Due to the complex nature of liver regeneration involving multiple pathways, redundancy may occur, whereby the absence of LPS may be compensated by other pathways; hence, gut microbiota may just partially contribute to the process. Our data also indicate that the interaction between gut microbiota and hepatic matrisome may play a crucial role in liver regeneration, particularly in the hepatic remodeling phase.

## 6. Limitations of the Study

A lack of correlation between mRNA and protein expression was observed in this study. This could be explained by various factors. Firstly, liver regeneration is a dynamic process and is spatiotemporally determined. Therefore, there is a possibility that protein synthesis might have been delayed, leading to differences seen in mRNA expression and protein concentrations. Secondly, regulation of synthesis and degradation rates may also affect the variability in protein concentration, which can be independent to the corresponding mRNA levels.

## Data Availability

The data on matrisome genes are available here https://docs.google.com/spreadsheets/d/1FOrT0VjSKH0QsJUQLv9ahYlEdGTaueK6/edit?usp=sharing&ouid=114940638212420284312&rtpof=true&sd=true (accessed on 18 April 2023).
